# Notes of a Visit to the Public Lunatic Asylums of Scotland

**Published:** 1856-07-01

**Authors:** John Webster

**Affiliations:** Physician to the Scottish Hospital


### Art. III.—NOTES OF A VISIT TO THE PUBLIC LUNATIC ASYLUMS OF SCOTLAND.

BY JOHN WEBSTER, M.D., F.R.S., AND F.R.C.P.,
*Physician to the Scottish Hospital.*

(*Concluded from page* 202.)

### Royal Asylum of Aberdeen.

The remaining establishment to which I propose directing attention, is that of Aberdeen,—the oldest Asylum in Scotland, with the exception of Montrose. Having been founded originally in 1800, upwards of half a century has elapsed, during which many benefits have been conferred upon afflicted lunatics belonging to the northern counties: since it was then, as now, the only large institution of the kind within these districts. The Asylum is situated in the immediate vicinity of Aberdeen, towards the north-west: where it occupies a pleasant, open, and airy position. The site lies somewhat low, with moderately elevated ground in the rear; nevertheless, it is considered salubrious. Having gradually increased from an hospital intended originally for about 50 patients, to an average now of about 350, the present structure displays correctly, in stone and lime, the progressive advances of the idea entertained by most persons regarding a receptacle for lunatics during the past half-century; and, as such, or historically, it amply merits examination by psychological antiquarians.

Various courtyards are attached,—fourteen in number,—ten of which being large, they supply ample means for classification. There are, besides, smaller courts for seclusion; so that, when it becomes necessary to separate any patient from other inmates, this can be accomplished out-doors, and in the open air, instead of placing the lunatic in a dark, confined, and often ill-ventilated, objectionable apartment, which I have sometimes noticed elsewhere: of course, the above proceeding becoming only advisable during suitable weather. The absence of long noisy galleries for day occupations at this Asylum, and the various suites of sitting-rooms, likewise merit approval; the only galleries used at present being bed-room passages, or corridors, which are never occupied during daytime, but constitute merely chambers of communication. It should further be mentioned that, the second-floor of this institution is solely appropriated for sleep-rooms, or private apartments: whilst there is no third story, unless in a very small division of the building. Again, noisy and dirty patients sleep in those parts of the Asylum which are single-storied, but not sunken, or lower than neighbouring constructions.

Some disadvantages should, however, be noticed in the oldest portion of the structure, which is built on a plan not properly admitting of extension; and whereby the kitchen, for instance, seems to have been stuck upon, rather than developed from, the original centre. The dormitories in some parts, also, are not admissible of being always conveniently classified, through proximity to the day-rooms occupied by that particular section of patients, to which these apartments are specially attached. Hence, inconvenience occasionally occurs when the inmates are retiring to bed; whilst this arrangement tends, likewise, to separate the attendants' sleeping-rooms from those of their patients. Besides such minor defects, I might add that, the earlier additions made to the building do not appear to have been conceived with much prospective sagacity. They seem badly placed, have an inferior appearance, and are on a lower level than the main structure: whereby, these succursals have formed rather an obstacle to, instead of an advancing step towards, subsequent improvements. Having been now so long in operation—since the Asylum was first opened—as also from the ordinary effects of time and constant use, a censorious critic might say, there is rather a want of smartness in some portion of the house furnishings, which looked as if the worse of wear, and would be improved by modernizing. However, several appendages are really of a superior description; and various extensive alterations, with improvements, being now in active progress, very soon the institution will possess additional accommodation for fifty more patients.

A new chapel has likewise been recently finished. Farther, the bathing apparatus is excellent, and much superior to similar appliances I have sometimes observed elsewhere. The separate sleeping-rooms are numerous, and by no means so large as the dormitories usually met with in other places: six to eight beds being frequent, but never more than eighteen occupying one apartment; whereas, in some similar apartments previously visited, twenty-four and twenty-six beds seemed not uncommon. All the sleeping-rooms for patients are upstairs: the ground-floor being, as already stated, appropriated solely as work-rooms, apartments for meals, or recreation. The whole appeared clean and well arranged. Hyper-critics might doubtless object to such dormitories upstairs being in any way intersected by a narrow passage running along the middle of a wing, or main building: the rooms for sleeping being on each side. Ventilation must certainly then be rather difficult to effect in apartments so arranged, whilst noise or disturbance may be thus more readily produced. Still, to my apprehension, this arrangement did not appear a really serious defect; and seeing much more accommodation was thereby obtained,—always an important object in reference to paupers, or those who pay a moderate sum for accommodation,—the plan had various advantages.

Although not altogether a professional question, some allusion to the financial condition of this Asylum deserves, at least, a passing remark: the facts being somewhat different from statements occasionally made in other quarters. Notwithstanding the low rates at which lunatics of the pauper class are kept in the Aberdeen institution,—viz., 15*l.* per annum, for every charge—board, lodging, clothing, and bedding,—there usually appears a balance at the end of each year. For example; upon an income of 5564*l.* 2*s.* 4*d.* during 1854, the treasurer reported that 305*l.* 14*s.* 9*d.* remained above the expenditure. Hence, this Asylum is self-supporting; whilst, it should be further remembered, such results occur without the assistance of annual subscriptions, donations from public bodies, or any appeal whatever to general charity, being thus quite different from what often occurs in England.

One important feature in the general arrangements of this establishment deserves special approval,—namely, the invariable rule which prevails throughout, of leaving the doors of every day-room, communicating with its appropriate airing-court or flower-garden, always *open* during day-time. Consequently, inmates have always free access to the one or other, as their feelings or wishes may incline. In this way, no appearance prevails of confinement; and patients may sit in the room reading, can walk about in the open court, or quietly repose in the shade, as best pleases each individual. There is no opening of door-locks, at particular times, to allow occupants to walk out, as if at drill: but obliged soon afterwards to retrace their steps, like some squad of prisoners. Nothing of the kind here exists; and I must repeat, this system of real non-interference seemed both beneficial and highly commendable. Of course, attendants were always present: whilst the judicious classification of patients became essential, in order to carry out this excellent scheme successfully. Somewhat similar arrangements are pursued in several French asylums I have visited: and partially, also, in some British institutions; but at no institution for lunatics, to the best of my recollection, was this plan of never locking-up lunatics within their own day-rooms, or airing-courts, so systematically adopted, as in the Aberdeen Asylum, for which the managers deserve much praise. Truly, so good an example ought certainly to be followed in other insane establishments.

When perambulating various precincts, the inmates generally appeared orderly, quiet, and clean in their outward aspect. The females seemed even less violent than usually noticed in similar establishments: where they are often noisy, and always got more excited on seeing strangers, compared with men placed under similar circumstances. The physical health of residents was reported very good, and few were confined to bed by bodily illness, especially among male patients: the actual number of sick persons being two males and five females, of whom only two had become seriously indisposed. Five males were placed in temporary seclusion within their own apartments, besides one female; and two others were likewise separately confined, or rather kept, for a time, in an open court, solely appropriated to excited patients: both being then exceedingly violent. One male lunatic was also similarly treated; but none of these individuals suffered under any kind of bodily restraint; their limbs were free, and they could walk about, lie down, or sit, as best suited; whilst, the weather being fine, and the courtyard airy and open, whatever exercise they might then take proved beneficial. Non-restraint characterised the system here pursued, and the strait-waistcoat was unknown.

During my last visit to this asylum, along with Dr. Macrobin, the physician, who kindly conducted me everywhere, and to whom my best thanks are justly due for his courtesy, both then and on the previous occasion, I was somewhat astonished, but after an explanation, rather amused, on finding two unexpected occupants in the seclusion court appropriated to excited male lunatics. How the parties got there, or who they were, at first seemed mysterious, as no one could speak respecting their individuality; and the two lads—for such they were—being so frightened, they could scarcely give an account of themselves. Thinking they might find fruit on the inner wall—which looked like that of a garden—the culprits had scrambled up to seize their anticipated prize, but tumbling over, thus got caught, as if in a trap, without any chance of escape. Lunatics sometimes run away from an asylum, but sane persons seldom break into such establishments, being always more anxious to remain outside, than to get within such enclosures. Henceforward, it will be so with these reckless youths, whom we released from durance vile, after a suitable admonition, carefully to avoid falling again into similar scrapes, lest they might be next time treated less mercifully.

Owing to the deficiency of accommodation for lunatics, in the northern counties of Scotland,—there being at present only a small establishment at Inverness, with another at Elgin, for pauper lunatics,—and from the frequency of mental derangement, especially throughout Highland districts, applications for admission into this asylum are always numerous; more so, indeed, than the executive can conveniently receive. Last year, for example, while sixty-five new cases were admitted, about forty had to be refused: solely on account of wanting the requisite accommodation, at the time of application; and even although some temporary dormitories had been erected, in order that the public convenience might be secured by every means possible.

At the time I visited this asylum, the aggregate lunatics amounted to 279: of whom 133 were male and 146 female patients. General paralysis comprised 4 males and 2 female inmates; the dirty cases amounted to 32, the sexes being equally divided; and lastly, several were complicated with epilepsy—the female victims of that intractable form of disease being, moreover, least numerous. Like other similar establishments, the various examples of amentia, mania, dementia, monomania, and melancholia became easily recognised. Hence, the patients actually under treatment presented nothing very unusual, many cases exhibiting the average type met with in most institutions for the insane; whilst a number were of such a chronic character, and had been so long afflicted by their mental malady, that any prospect of ultimate recovery was considered almost, if not utterly, hopeless.

Amongst the females noticed during my inspection, one patient particularly attracted attention, in consequence of her un-feminine appearance. In fact, she looked rather like a man than a woman, although dressed in female attire. Indeed, persons of a fanciful imagination might have even considered they then saw a Crimean heroine, as her face was decorated with large whiskers, beard, and moustaches—the last full grown! Having quite the masculine aspect, and being altogether a more curious specimen of the kind, than I had ever previously remarked, an inquiry was consequently made respecting her history. This maniac first became an inmate in 1845, being then aged about 45, and married, but without any family. Previous to admission, she had been ill nearly a year, and gradually become insane. No exciting cause was assigned, but domestic unhappiness had been presumed. At first, she laboured under melancholia, and then attempted suicide by cutting her throat. After admission, the patient appeared to entertain high religious fancies, having ideas about seeing Christ, with similar delusions: but at present, is fond of dressing in imitation of royalty, and often calls herself the "king," the "queen," and so forth.

This unfortunate creature being a parochial lunatic, sent from a distant part of Scotland, and as no friend or relative now ever visits the asylum, it became impossible to obtain farther information than the above meagre details here given respecting her former condition, or hereditary features. Nevertheless, so well marked an example of a bearded female at least deserves record, on account of the great rarity of similar hirsute women.

On the last visit I made, in order to get better acquainted with the varied arrangements, and excellent system pursued at this well-managed establishment, every favourable impression previously received was confirmed, rather than shaken, by farther acquaintance. The following memoranda, then written, will indicate my opinion upon that occasion: "Arrangements are good, and system pursued is generally excellent. Many efforts appear made to improve the institution, as also to ameliorate the condition of its inmates. In the court-yards and day-rooms numerous tranquil patients were waiting to frequent chapel—to-day being Sunday. Altogether, I think more favourably of this asylum than previously, and it seems to improve by farther examination." Such were my observations then recorded; and they are here quoted to express the sentiments I entertained on that occasion.

During the twelve months, ending the 31st March last—comprising the official year of this establishment—65 new patients were admitted, 21 being males and 44 females, or almost two-thirds of the whole; thus indicating greater prevalence of insanity amongst that sex, than the opposite. The number discharged cured were 11 males and 28 females, or 39 altogether; which gives a ratio of 60 per cent. to admissions. The deaths amounted to 19; consisting of 14 male and 5 female inmates; hence making the rate of mortality 29·23 per cent. similarly calculated. It should, however, be mentioned, as rather remarkable, the proportion of deaths amongst male patients greatly exceeded that of females: the amount in the former sex being so high as 66·66 per cent.; whereas, the latter ranged 11·36 per cent. or about one-sixth.

Respecting the forms of disease manifested by patients when admitted, mania appeared the most common; almost half being of that category. Dementia and melancholia ranked next in frequency; both these varieties having each supplied nearly one-fifth, respectively. Three cases were complicated with epilepsy—all females; and 2 males, labouring under dementia, also suffered from general paralysis. Amongst the causes assigned as exciting insanity, intemperance occupied a prominent influence: which, unfortunately, too often proves the case elsewhere; the sexes being about equally divided. Hereditary predisposition embraced upwards of 38 per cent.: which seems a high proportion, but is influenced, doubtless, by the circumstance that many new patients belonged to Highland districts, where hereditary taint seems often transmitted; and lastly, a rather larger proportion of the female patients, than is sometimes observed, had become insane through childbirth and nursing. Amongst the 19 deaths recorded, 12 of the whole number, or nearly three-fifths, were examples of dementia, only 2 of these being females; whilst it is important to add, paralysis proved the immediate cause of death in half the fatal cases last specified. Of the patients cured, mania constituted by far the highest proportion: 26 recoveries being of that description, of whom more than half had not been attacked by their mental malady beyond one month prior to admission; thereby demonstrating the great advantage of early removals to an asylum, and thus obviate many sad results which so often arise from detaining recently affected insane patients at their own homes. An opposite proceeding is always much to be deplored, especially amongst pauper lunatics. Knowing the erroneous notions which may often prevail upon this point, in some districts, through ignorance, prejudice, and even interested motives, on the part of parochial authorities, the above important fact, in reference to early treatment, cannot be too extensively promulgated, seeing it coincides with the uniform experience obtained at all public institutions for the insane. This gratifying result should be more particularly disseminated throughout Highland counties: where intelligence appears lower, and prejudices stronger, upon this question, than among more southern or commercial communities.

Amusing and occupying the patients are assiduously carried forward. These it is unnecessary to specify in any lengthened paragraph: the proceedings in this respect being satisfactory, and similar to other well-regulated asylums. The females find ample and varied occupation in domestic routine, or in other employments. Many male patients are generally engaged under the direction of gardeners, and the superintendence of attendants, throughout the enclosed grounds appertaining to this institution, which altogether contains about twenty-four acres—nearly six being covered by its different buildings, or appropriated as airing courts. From nine to ten acres are cultivated as gardens, mostly for kitchen produce: whilst about eight acres—now let on lease—or occupied by constructions not forming part of the institution—are capable, in most part, of being brought within the hospital enclosure.

Notwithstanding the above appliances, it is considered desirable, in order that out-door occupation may continue adequate to the increasing number of field and garden labourers, that the extent of ground available for their operations should be enlarged. It is wished likewise, further to augment the means of in-door employments amongst male patients, which becomes usually more difficult of accomplishment than with most female inmates of lunatic establishments: particularly, where patients belonging to the middle or upper classes of society are admitted; this being much more frequent in the public asylums of Scotland than elsewhere.

The extension of various buildings and alterations, at present in progress, have curtailed materially the personal comfort of residents. Despite all these interferences with the ordinary movements of such an institution, no untoward circumstance has, however, resulted, excepting that recently, a convalescent female patient—abusing the trust placed in her recovered sanity—took advantage of an imperfect barricade, and escaped to her friends; who chose, instead of returning, to retain their relative. With reference to the extensive improvements in course of completion, I cannot do better than now transcribe the following paragraph from the recent Annual Medical Report, which says:—

"The compensating advantages that will immediately result to the residents themselves from these operations will be more than adequate to the discomfort undergone; for while the external appearance of the establishment will be improved, and its extent considerably enlarged, an inferior description of apartments will have been re-placed by rooms more in harmony with the newer portions of the building; several additions made and alterations effected admitting a more detailed classification of inmates, a better style of accommodation afforded to private patients, and various desirable improvements carried out in domestic arrangements."

The medical officers of this asylum consist of Dr. Jamieson—resident physician and superintendent, with Dr. Macrobin as consulting physician: the latter being non-resident. Besides these able and accomplished practitioners, whenever any grave surgical disease supervenes in a patient, further assistance is always procured. This occurred recently, when Dr. Keith—senior surgeon to the Royal Infirmary, and distinguished for his practical knowledge—was called upon to give valuable assistance in two cases of importance. Another excellent feature characterising this asylum also deserves much praise—namely, medical pupils are admitted to attend the physicians, in order to obtain information and clinical experience respecting the treatment of mental diseases. In pursuance of this principle, and to make the attendance of professional students more useful, Dr. Jamieson gave a course of lectures on the medical jurisprudence of insanity. These he afterwards published: and all were, I can add, very favourably received by the profession. Consequently, whether the benefits conferred upon suffering humanity, or the diffusion of sound knowledge thus promoted be considered, much credit is unequivocally due to the staff of this establishment: and therefore I would say, with much sincerity:—

O! si sic omnes.

### General Remarks.

Reviewing the aggregate statements contained in previous pages, it there appears that, at the period of my visits to the different asylums, during the months of August and September, the total population then resident in the six public establishments inspected was considerable. To place the whole under one view, as likewise to show the general movement of patients, throughout the past twelve months, at these institutions, I have constructed the following table, to which attention is now requested:—

*Table showing Population and Movement of Inmates, at Six Public Asylums for the Insane in Scotland, during Twelve Months. Compiled by Dr. Webster.*

| Asylum. | No. of Patients. | | | Admitted. | | | Discharged cured. | | | Deaths. | | |
|---|---|---|---|---|---|---|---|---|---|---|---|---|
| | M. | F. | Total. | M. | F. | Total. | M. | F. | Total. | M. | F. | Total. |
| Edinburgh | 273 | 283 | 556 | 98 | 114 | 212 | 28 | 66 | 94 | 24 | 27 | 51 |
| Glasgow . | 199 | 182 | 381 | 123 | 117 | 240 | 60 | 56 | 116 | 32 | 30 | 62 |
| Perth . . | 77 | 64 | 141 | 23 | 13 | 36 | 6 | 11 | 17 | 7 | 8 | 15 |
| Dundee . | 94 | 113 | 207 | 26 | 25 | 51 | 12 | 9 | 21 | 6 | 5 | 11 |
| Montrose | 96 | 133 | 229 | 39 | 52 | 91 | 18 | 19 | 37 | 11 | 10 | 21 |
| Aberdeen. | 133 | 146 | 279 | 21 | 41 | 62 | 11 | 28 | 39 | 14 | 5 | 19 |
| Total . . | 872 | 921 | 1793 | 330 | 362 | 692 | 135 | 189 | 324 | 94 | 85 | 179 |

According to this document, it appears the total lunatic inmates amounted to 1793, the majority being females: of whom the number was 921, against 872 males. However, when the western districts of Scotland are compared with the eastern, the portion of each sex labouring under insanity seems very different. In the Glasgow Asylum, and that of Perth: which, although occupying a central position, is still more western than otherwise, in reference to the patients admitted, it will be perceived male residents were most numerous: seeing, these two institutions then contained 276 males and 246 females. On the other hand, at Montrose and Aberdeen the reverse prevailed: their population being 229 males to 279 females. At Edinburgh, also, on the east coast, females likewise preponderated. Again, in reference to new admissions, mental diseases seemed certainly to occur oftener among men, belonging to the western counties of

Scotland, than to the eastern: where 146 new male patients were admitted, against 130 females; whereas, 60 males to 93 females—all being recent cases—were received at the Montrose and Aberdeen Asylums. From such data, it may be fairly inferred that madness prevails more frequently in the western counties of Scotland amongst males: whilst, on the east coast, females oftenest become afflicted with mental alienation.

Another interesting feature should also be noticed in this table—viz., more females than males were cured; whereas, the deaths amongst male patients exceeded in amount those recorded throughout the other sex: thus showing that insanity proved more curable and less fatal in female, compared with male lunatics. These results are instructive: and should they be supported by further experience, especially if illustrated by a large array of additional facts, psychological physicians could then deduce important conclusions respecting the comparative frequency and prognosis of mental maladies when affecting the two sexes, with much greater confidence than heretofore.

Recent investigations having proved that the total lunatics and idiots now chargeable to parishes exceeds 3600: besides numerous private patients, of whom many are at present inmates of public asylums; it consequently appears, there is by no means adequate accommodation for the large number now afflicted with mental disease, especially in the northern counties. Hence, both at Montrose and Aberdeen, petitions for admission have been for some time back much more numerous, than these institutions could accommodate. When I visited the above asylums both were more than full, every exertion having been made to receive urgent and recent cases, wherever the executive could admit such sufferers. The patients refused admission during last year were considerable; and during my second visit to the Aberdeen Asylum, it was found impossible to take charge, even temporarily, of a sailor lad, who had become, only a few days before, furiously insane. Such statements demonstrate the urgent necessity for another asylum in the northern counties of Scotland: more imperatively, as from thence many applications are constantly made for admission into the two institutions just named. The authorities and landowners, both of the north, and in the western islands, ought therefore to bestir themselves in order to remedy this deficiency: particularly, when they know how important it becomes to place insane patients immediately under judicious treatment. As these advantages cannot be obtained at home—certainly by poor Highlanders—and seeing madness unfortunately prevails to a considerable extent within the districts mentioned, such want of proper accommodation consequently becomes more lamentable, and urgently demands an efficient speedy remedy.

Besides previous cursory observations respecting public Asylums for the Insane in Scotland, I would add some remarks in reference to their present management, as also on several points which deserve discussion. These are neither of magnitude nor importance: since most Scottish institutions deserve commendation in many essentials, and have kept pace in the onward march of improvement, fully commensurate with modern civilization. Nevertheless, all cannot be held up as models for imitation in every respect. Nay, according to my humble judgment, some appeared open to criticism in one or two phases, which consequently require amendment. The observations about to be made will, however, I hope, be received in the sense they are intended —namely, like mere suggestions expressed solely with a view to renovate, as it were, those defective movements which are occasionally observed in old physical constitutions, or antiquated corporations.

However, before entering upon the points subsequently mooted, I must premise that, the medical officers of every institution visited, seemed all actuated by the utmost zeal to promote the welfare and comfort of those afflicted human beings committed to their superintendence. Should defects exist, it is not their fault if these continue unremedied. Errors of construction are not easily amended. Mistakes in legislation, or erroneous rules relative to general management, they cannot always correct; seeing laymen sometimes improperly interfere, even with medical questions. Still, it would be unjust to deny that, generally, Asylum-managing committees are actuated by the same benevolent motives influencing resident officers,—namely, to advance by every possible means the material comfort of inmates, and to improve the institutions under their control, by adopting judicious amendments.

Regarding the official staff in lunatic establishments, it struck me, however, when considering the matter, that at several, some change in their position might be made most advantageously. For instance, the resident medical superintendent should have more administrative power than he often possesses, and should be better remunerated than sometimes happens. He ought to exercise *paramount* authority in everything appertaining to the management, and moral, medical, or physical treatment of patients. He should likewise attend all meetings of managing committees, although without the privilege of voting—from being a salaried officer—in order that he might give his opinion respecting the admission of new patients, or upon any professional questions which then arose: as also to prevent any future misunderstandings. The matron—who is sometimes too highly salaried, in relation to other officials and her actual position—appears frequently not sufficiently subordinate. This objection has been felt elsewhere; and in France, for example, where they manage many things often so well in lunatic asylums, a lady matron is almost unknown. Throughout Scotland, as also in England, sufficient attention is not invariably paid to their qualifications in the character of housekeepers, head attendants, and as sick nurses; when the governors select for appointment this occasionally rather too self-important personage.

Some institutions have consulting physicians; but other establishments are without such medical attendants. The system should be uniform: and there ought invariably to be both a consulting physician and surgeon, whenever possible. These officers should be called in consultation respectively, at the discretion and request of the medical superintendent: for which duty they ought to be remunerated liberally. There should further be always two resident medical officers in every asylum; one being the assistant, and subordinate to the resident physician. Besides which, but particularly at large establishments, there ought to be resident pupils, or "internes," as usually prevails in France. This constitutes one of the many good features characterising various public insane asylums of that country.

Every building for the reception of lunatics should be disconnected with any other public establishment, whether infirmary, dispensary, or poorhouse. Even in lay management, it is desirable there should be no kind of union: much less any physical conjunction. Wherever this system exists, it ought to be altered as unsuitable; from being apt to become, in various conceivable ways, disadvantageous to the lunatic institution so situated.

The two departments for private and pauper patients—very common in the public asylums of Scotland, as likewise the accommodation supplied in each of these divisions, should be properly distinguished, and always separate. Farther, the classification of inmates ought to be made, in the first place, more with reference to the phase of their mental malady, and less as to the pecuniary allowance received. This desirable object may not be always possible in limited or old constructed dwellings; but henceforward, at every new asylum, which shall admit patients belonging to various social grades, special attention ought to be directed towards attaining separate buildings, like those now at Morningside, having gardens attached: instead of making—which occasionally happens—a common class, composed of the poorest private patients and pauper inmates. In truth, the educated and refined should never be indiscriminately mixed with the debased and unpolished, when afflicted by such a calamity as poverty, conjoined to mental alienation. I would further remark, that the impression produced on my mind, whilst visiting particular asylums was, the distinctions adopted amongst patients sometimes appeared too much based on a system of money classification—each inmate deriving advantages according to their respective payments. Hence, individuals paying similar rates, although in a different mental condition, were often associated together, irrespective of their nosological peculiarities.

At an asylum I lately visited abroad, a totally opposite method was adopted: but which, however, carried the treatment too far the reverse. Patients in that establishment were usually classified according to their diseased mental condition, so that inmates paying a high board became associated with those of a lower scale; the chief advantages obtained by the higher-paying classes being a better kind of fare, in the same dining-hall with the others, and from having superior furniture in their private apartments. This mode of classification, though better in some respects than the Scottish system, has, nevertheless, a tendency to depreciate the condition of the upper class, by making them live, while in a similar mentally weak condition, with persons often of inferior education, of different habits, and varied acquirements. Both plans seem disadvantageous; but a combination of the two would be followed by fewer objections, in comparison, with the one usually adopted in North Britain.

At those Scottish asylums, where an inmate's board is paid quarterly, and in advance, some relaxation ought to be made in regard to the stringent rule now in operation, with reference to these money questions. It may be often very proper, when a new patient enters, that the first payment should be anticipated; but it looks rather like sharp dealing, if not implied injustice, wherever a rule exists like the following:—

"When any patient is removed, or dies before the close of a quarter, the committee shall have power to decide whether any, or what portion, of the sum advanced for board may be refunded."

Such legislation seems, at least, unworthy of all respectable institutions: and in these cases an equitable proportion ought to be refunded as a matter of right, without, of course, any petition or formal application by relatives—especially if they are in poor circumstances.

So much of all pre-paid boards as did not seem fairly required for the time passed in the asylum, up to a patient's death, should be returned if demanded. Carrying into effect the proviso now quoted, sometimes proves very annoying to officials and others, who come in contact with the friends and connections, especially of deceased patients; whilst in some cases it may cause—as, for example, with convalescents—the patient being removed too quickly, owing to the arrival of quarter-day. Besides, it may even occasion a longer residence than is desirable, in consequence of three months' farther board having become payable.

Instead of the above proceeding, an invariable regulation should prevail, that a patient's discharge may take place at any time thought advisable by the resident medical superintendent, without pecuniary loss arising to the party, but not according to the practice now prevalent: namely, at the end of each term. The board of private patients should likewise be paid as it is charged—viz., by weeks, and not quarterly.

Prior to admitting a lunatic into any public asylum, some relation or friend entitled to perform such acts, must present a petition to "The Honourable" the sheriff of the county, wherein the asylum is situated, or to his substitute, which "humbly showeth," the afflicted person there named is in such a state of mental derangement, as to require treatment in a lunatic institution. This document must be accompanied by one medical certificate. Having considered such petition, the sheriff may at once order the party's admission. In short, this formality—for it is virtually nothing else in most cases—treats the maniac like an accused pannel on trial, instead of a suffering invalid afflicted by mental disease, requiring medical aid and benevolent superintendence: certainly, not a judicial decision thus promulgated as if by some legal tribunal. Unless in reference to delinquents contravening the laws of property or persons, similar applications to sheriffs should never be required. Proper medical certificates are all that is necessary, in ordinary cases of insanity: and upon these managing committees should alone decide.

When any criminal lunatic is consigned to a public asylum, then a judicial warrant becomes essential: but otherwise it seems superfluous, and may be discontinued. In England no authority of this description is essential; where, it cannot be asserted any evil consequences ever ensue, because lunatic patients are admitted, for instance, into Bethlem Hospital, without the sanction of a judge, or even the police magistrate. As controller and official visitor of every lunatic establishment, within his own jurisdiction, there could not be a more appropriate supervisor than the sheriff: who, being the highest legal authority in every county, ought therefore to possess that power to its fullest extent. Such supervision becomes most beneficial. Nevertheless, believing the sheriff's warrant unnecessary, excepting in criminally accused lunatics, I consequently think there should be invariably *two* medical certificates, stating far more minutely than at present, not only the opinion of the gentlemen signing, but the chief symptoms inducing them to conclude the party is insane: and that not so much from mere belief, as actual personal knowledge. These particulars every medical practitioner should certify like an ordinary declaration, and in common language, not "*on soul and conscience:*" which words the printed form used for that purpose constantly contains. Feeling great repugnance to employ similar expressions, when writing medical opinions, and thus taking an oath, whilst performing only ordinary professional duties, I would strenuously advise the phrase now quoted to be expunged by future legislation: considering it both misapplied, and highly objectionable. This opinion refers quite as strongly to ordinary certificates, which physicians or surgeons are sometimes called upon to give, respecting the health of persons they may attend professionally: since these documents must likewise contain the phrase "soul and conscience." In such cases it ought to be sufficient, if the person signing certifies any fact, like a man of honour and a gentleman. He always expresses truth on every occasion, whether simply by word of mouth, or in *litera scripta.*

My chief object being, when drawing up these Notes, to give a brief account of the asylums inspected and their present condition, rather than to discuss minutely the laws affecting lunatics, it may perhaps therefore appear somewhat out of place now to enter into any lengthened disquisition upon the latter subject; still, in the early portion of this communication, having enumerated the Acts of Parliament by which these establishments are governed, and how the insane should be treated, speaking in a legal sense, there is one point in reference to single lunatics deserving consideration. I here allude to the clause in the statute of George IV., whereby it is enacted that "no person, *except a relative*, shall receive any one insane patient without a sheriff's order and certificate." According to this enactment, relations may retain lunatics at home, and thus exempt them from legislative interference. To show the manner this exemption occasionally works, one interesting case that recently occurred in a rural district of Scotland may be mentioned, and of which I am cognizant, having myself seen the individual. The subject referred to was a young man, in whose family insanity seemed hereditary. Having become attacked with mania, the relatives sent him to an asylum: where, after a short residence, his attack became much ameliorated, and ultimately the improvement appeared so decided, that he was removed home. There, however, having soon got worse, his father and brother being unable to control this now furious maniac, and at the same time to attend their own out-door occupations, generally tied the poor fellow with ropes, then laid him on a bed in an empty room, and often on his back in the garden, where he remained until they returned in the evening. This cruel proceeding becoming notorious in the neighbourhood, a benevolent gentleman took up the case: but he encountered great difficulty in persuading the parent and brother either to alter their conduct, or to send the unfortunate lunatic again to an asylum. No law could compel these parties. No stranger, nor even a medical man, possessed the right to interfere. This patient had never committed any criminal act; and none of the local magistrates were made aware of the circumstances. In short, nothing could be done without meeting many obstacles: until at last, both by persuasion and even threats on the gentleman's part—already mentioned, this ill-used, most violent, and dangerous maniac was again replaced under proper management in a public institution. Were the laws more stringent respecting single patients, painful cases like the above would then be more easily remedied, than at present seems possible, according to legal formalities.

Other cases illustrating the baneful effects of too early removals of insane patients from asylums might be mentioned, but it appears supererogation. However, before leaving the subject, I would observe, that such proceedings are much more frequent than seems advisable; as may be shown by the following statement obtained from one of the institutions previously passed under review. During a period of nearly two years, 45 males and 50 females—altogether 95 patients—were discharged from the asylum in question. Of these, 26 male and 29 female inmates were dismissed in accordance with the medical superintendent's advice; while 19 male and 21 female patients were removed contrary to his recommendation. Amongst the former class—55 in number—6 were transferred to other institutions, being only improved; whereas, the remaining 49 appeared cured—using the term as merely indicating their greater or less ability to perform ordinary duties of life. The results of those discharged as convalescent were thus reported. Not known, 9; returned, 7; but of whom some were relapses, having been in the house more than once previously; and 2 males were marked as doubtful, in reference to their recovery. Consequently, there remained 14 males and 17 females, or actually 31 cases out of 38, whose ultimate history could be accurately traced: making 81 per cent. who became perfectly cured. Again, of the 40 patients removed contrary to medical advice, 13 were discharged under protest, and 27 through the influence of persuasion. Amongst the latter description, most were parties who then seemed either convalescent, but not sufficiently tested: or those who, from the form of their mental disease, appeared safe and manageable. However, with reference to many of these individuals, the entreaties of relatives, or inspectors of poor, became much more readily listened to, in order to diminish over-crowding, and to make room for the more clamant cases.

Respecting the 27 patients reluctantly discharged through persuasion, 10 seemed convalescent, and 17 were in various stages of improvement. Of the above number, the result stood thus:

6 remained unknown, 6 continued improved, 7 were considered convalescent, and 8 soon became much worse: of whom 3 died, and 2 returned to the asylum, while 3 still enjoy their liberty. Amongst the 13 patients removed under protest, only 1 could be considered as really convalescent: though all the others, except 1, had made more or less improvement. In these cases the following results were reported: 2 could not be ascertained, 4 remained the same, while 5 soon got worse; and lastly, 2 died—1 being by delirium tremens. This fatal case constituted an example of dipsomania in a well-educated young man, who came under treatment as a suicidal melancholic. Nevertheless, he was removed from the institution, about a month after his entry, in opposition to most earnest entreaties and warnings respecting consequences. Thus, of the parties discharged contrary to advice, and whose subsequent history is known, none have improved: whilst the majority afterwards either became worse, or ended fatally. These facts are instructive, and indicate conclusively the injurious results frequently attending premature removal of lunatic patients from asylums, when only approaching a state of convalescence.

Notwithstanding the brief remarks made in a former page, apparently censuring some regulations now in force at particular institutions: readers should, however, always remember that it was in Scotland where one of the earliest public asylums for the reception of lunatics, and improved treatment of mental diseases, was first founded, throughout the entire United Kingdom. Besides this creditable distinction, when contrasted with other countries, it should likewise be recollected that of late considerable progress has been made in their management, highly honourable to many official functionaries. Indeed I can justly add, in addition to occupying, amusing, and physically treating insane residents, in a manner very superior to the system pursued in former times, the intellectual culture of such patients—often previously much neglected—has been materially advanced: not only greatly to the lunatics' present comforts, but also their future advantage, when discharged from the asylum. Hence, these proceedings reflect considerable credit on the gentlemen by whom such praiseworthy tasks—often most beneficial—are undertaken.

Believing some remarks upon the regulations whereby public asylums for the insane throughout France are at present governed, and on the laws affecting lunatics: as likewise, the proceedings which become necessary prior to any patient being admitted into such establishments, before concluding these Notes, one or two brief observations will, it is hoped, be neither considered out of place, nor irrelevant to the legal questions I have discussed, in various paragraphs of this communication.

Therefore, I would now observe that, according to the Act of

30th June, 1838, each department of France is obliged to provide a public establishment, especially destined for receiving and treating lunatic patients belonging to the district; or to arrange, under the Minister of the Interior's sanction, with a public or private asylum in the same or a neighbouring department, to admit their insane paupers. It is, however, permitted in certain cases to appropriate a separate division, in civil hospitals, for lunatics, should there be sufficient accommodation for not less than fifty patients. As every lunatic establishment is now placed under the direction of the Préfet of the department, the President of the Tribunal, the local Procureur Imperial, Judge of the Peace, and Mayor of the Commune, and as the institution must be visited by the Procureur of the Arrondissement, at least every six months (in addition to visits made by the Préfet, with any other official persons delegated by him, or the Minister of the Interior), there is some guarantee the asylum will be properly conducted. Besides these regulations, before an establishment can be opened for the admission of insane patients, all rules for their internal administration must be approved by the Minister prior to being put in force. By another clause of the same Act, it is expressly forbidden for any person to establish, or even to superintend a private insane asylum, without government authorization; besides, in such cases, it is also enacted that, every house—intended for the reception of lunatic patients—should be entirely separate from any private establishment receiving inmates affected with other diseases. Lastly, the Procureur of the Arrondissement must officially visit every private asylum in the district: at least once in three months, at undetermined periods.

According to King Louis Philippe's ordonnance, of the 18th December, 1839—which regulates many details not comprehended in the previous Act of 1838—it is ordered, that every public asylum for the insane shall be administered under the authority of the Minister of the Interior and the Préfet of the department, assisted by a commission, acting gratuitously, of five members, and appointed by the Préfet. The Director of such establishment, as also the Physicians—both chief and assistants—in the first instance, are nominated by the Minister; but if vacancies afterwards occur, the Minister appoints from a list of three candidates proposed by the Préfet. However, some modification has been recently made respecting these employments, although in effect the chief patronage still remains with the Minister: since he may add certain parties, of his own free will, to the list of candidates, and then nominate his favourite to any vacant office. Besides, as the Minister may revoke all appointments of director and physicians, upon the report of the Préfet; as he settles the salaries of officers; and farther, seeing the Préfets are only Government servants, by whom they are appointed, and at whose pleasure they retain their offices, the Minister of the Interior becomes, in fact, the sole dispenser of every important appointment attached to the public insane asylums in France; much in the same way as the Minister of Justice possesses legal patronage. Although the chief physician must reside, according to this ordonnance, within the asylum, he may nevertheless, through favour, obtain a special permission from the Minister, to live elsewhere. In that case, however, he ought to visit the lunatics confided to his care, at least once every day: and, if prevented, this duty must be performed by a resident physician.

The above constitute some of the chief regulations respecting public insane asylums. But when any person is desirous of obtaining a licence to open a private establishment, such applicant must petition the Préfet of the department in which the proposed institution is situated, to whose satisfaction it should be proved that, he is twenty-one years of age, and in the enjoyment of all his civil rights: that his conduct and morals have been good during the three previous years—as shown by the certificate of the Mayor of the Commune in which the party has resided; and lastly, that he is a Doctor of Medicine. However, in cases where the petitioner does not possess that qualification, he may then produce an obligation from some physician who engages, with the Préfet's approval, to undertake the medical duties of, and to reside in, that asylum; but as the Préfet can at any time revoke this nomination, it is not likely the treatment of the patients will be much neglected. To these details respecting the constitution and ordinary government of public and private insane establishments throughout France, I will only add that, besides the official persons previously mentioned, there are also two Inspectors-General of all the lunatic asylums in the empire, with one adjoint Inspector: whose special duties, amongst others, are to visit and report to the Minister of the Interior, in reference to the management, or otherwise, of the insane; and everything of importance connected with these establishments.

By the present lunacy laws of France, there are two classes of patients in establishments for the insane: namely, 1, voluntary; and, 2, those designated *d'office.* Regarding the former, or voluntary patients, prior to being admitted into an asylum, a petition is presented to the authorities by some near relative of the party considered a lunatic. This document must be accompanied by the certificate of *one* legally qualified medical practitioner, who states the patient is insane, and requires confinement in an asylum,—the characteristic features and chief symptoms of the mental malady being specially mentioned. Besides these essential requisites, the medical certificate ought not be of longer date than fifteen days previously; and it will not be received, if signed by any relation of the patient: or, where the party signing is a medical officer in the establishment, to which the lunatic will be consigned. This document, however, may be dispensed with in very urgent cases, where the individual's safety, or that of the public is compromised: if remedied by subsequent proceedings. After the patient's reception, every paper respecting the case in question must be transmitted, within twenty-four hours, to the Préfet of the department.

In reference to judicial lunatics, or those technically classed *d'office* cases, the Préfet, and certain public officers, may order the admission into an asylum of any interdicted or non-interdicted person, considered as actually insane, in order to receive proper treatment. He may also similarly confine an individual, whose mental condition, or state of alienation, compromises public order, or the safety of the community. Further, in cases causing imminent danger to the public peace—provided the fact is attested by an authorized medical practitioner, or even from general notoriety—a commissary of police, or the mayor of an arrondissement, may place such dangerous lunatics under restraint; but these examples must be immediately afterwards reported to the constituted officials, who make additional inquiries, if deemed necessary, and act accordingly.

Such are the proceedings considered essential when consigning lunatics to an asylum. Previous, however, to that step being taken, some remarks respecting the procedure usually adopted with regard to private patients alleged to be insane, may not appear altogether supererogatory on the present occasion.

When any individual is suspected to labour under an attack of mental disease, especially if moving in the middle or upper classes of society, instead of proceeding as in England under similar circumstances, a *Conseil de famille* assembles, who see the party implicated, examine the whole case, and then draw up a *procès verbal* of the facts, for the *Procureur Imperial.* This judicial authority now orders two medical practitioners, authorized to perform such duties, to visit the party separately, take evidence, and afterwards forward him their opinions. On these documents that magistrate pronounces his judgment: when the patient is either sent to an asylum, or otherwise treated, as he may decide.

Although the above mode may be usually followed in ordinary cases, sometimes more summary measures become necessary with persons, in whom an attack of insanity has suddenly supervened. For instance, should a legally qualified medical practitioner think any patient, then under his care, is actually insane, and dangerous to others or himself, he may at once convey the party to a *maison de santé*, and there leave him along with a certificate of insanity, containing full particulars. The proprietor of the institution thus selected immediately forwards a statement of every fact therewith connected to the Inspector-General of Lunatics, who subsequently sends two physicians to examine the patient separately, and to report specially respecting the case, with its attending circumstances: upon which that officer makes his decision.

To illustrate the application of this latter form of proceeding, I subjoin an outline of three cases which actually occurred to a professional friend of mine practising in Paris. 1. A British peer called one morning to consult that gentleman respecting his health. Having described various symptoms, it became very evident the party had lost his reason. This suspicion became fully confirmed by the Noble Lord producing a bowie-knife, with which he threatened to kill an individual then named. After some parley, the physician induced his Lordship to take a drive, as if for recreation, and thus carried him off to a *maison de santé*, where he was safely lodged with a proper certificate. The inspector-general having been speedily informed of the occurrence, ordered two sub-inspectors to investigate and report their opinions. Every legal formality being thus complied with, and as the nobleman so confined was found to be unequivocally insane, he remained under treatment until discharged convalescent. 2. This instance occurred in an Irishman, who had taken the pledge to abstain from intoxicating drinks in Ireland. To quiet conscience, and not to violate his promise, a visit to Paris was undertaken. There, however, the individual lived so freely that delirium tremens ensued, which soon required medical attendance. As no doubt existed respecting the attack, or its appropriate management, my medical informant speedily transferred his patient, much in the same manner as the previous person, to a *maison de santé*, in which he was placed with a certificate of insanity properly filled up, and there he stayed till recovery. 3. The third example also occurred in the "*clientelle*" of the same practitioner. One evening he was sent for to a neighbouring café to see an Englishman, then labouring under delirium tremens, and very furious. Fortunately, Mr. Forrester, the London police officer, was also in the house: who, being accustomed to manage physically dangerous customers, seized this party under the physician's directions, pinioned him in such a manner as to prevent further mischief, and afterwards transferred him, then raving mad, to a *maison de santé*. There he also continued till cured: the proper formalities having been complied with, as in the other two cases just quoted.

No difficulty was encountered, nor did injurious delay occur in any of the three patients whose history has here been briefly related, to describe the procedure which can be legally adopted in France, when insanity suddenly supervenes: or, wherever persons so affected have become dangerous either to themselves or the community. In this way, no time is lost before placing similar examples under judicious superintendence. Consequently, in these cases, or when a *Conseil de famille* has assembled to investigate the condition of any alleged lunatic, whose mental affection appears doubtful, or seems of a more chronic character, much advantage often accrues from adopting either measure specified. Neither friends nor attendants are certainly so apt to suffer personal injury, where furious maniacs can be in this way easily, yet legally, transferred to places of safety, and thereby receive right treatment. Such proceedings prove, besides, always highly beneficial to the patient. Further, public scandal, and all unpleasant discussion respecting the private affairs or strange conduct—often originating from disease—of individuals, especially if moving in the upper classes of society, will be effectually obviated. This undesirable result rather frequently supervenes in England, when an investigation *de lunatico inquirendo*—the more likely if about a party possessed of property, or occupying an elevated station — becomes instituted before ordinary legal tribunals. Therefore, should alterations in existing lunacy laws be proposed, the above instructive facts well deserve mature deliberation.

